# The Impact of Multiple Species Invasion on Soil and Plant Communities Increases With Invasive Species Co-occurrence

**DOI:** 10.3389/fpls.2022.875824

**Published:** 2022-05-31

**Authors:** Dušanka Vujanović, Gianalberto Losapio, Stanko Milić, Dubravka Milić

**Affiliations:** ^1^BioSense Institute, University of Novi Sad, Novi Sad, Serbia; ^2^Institute of Earth Surface Dynamics, University of Lausanne, Lausanne, Switzerland; ^3^Department of Biosciences, University of Milan, Milan, Italy; ^4^Laboratory for Soil and Agroecology, Institute of Field and Vegetable Crops, National Institute of the Republic of Serbia, Novi Sad, Serbia; ^5^Department of Biology and Ecology, Faculty of Sciences, University of Novi Sad, Novi Sad, Serbia

**Keywords:** *Acer negundo*, *Amorpha fruticosa*, *Fraxinus pennsylvanica*, multiple invasions, native plants, riparian ecosystems, soil communities

## Abstract

Despite increasing evidence indicating that invasive species are harming biodiversity, ecological systems and processes, impacts of multiple species invasion and their links with changes in plant and soil communities are inadequately documented and remain poorly understood. Addressing multiple invaders would help to ward against community-wide, synergistic effects, aiding in designing more effective control strategies. In this work, correlative relationships are examined for potential impacts of three co-occurring invasive plant species, *Amorpha fruticosa*, *Fraxinus pennsylvanica*, and *Acer negundo*, on soil conditions and native plant diversity. The research was conducted in riparian ecosystems and included the following treatments: (1) co-occurrence of the three invasive plant species, (2) occurrence of a single invasive species, and (3) control, i.e., absence of invasive species. Co-occurrence of three invasive plant species caused higher direct impact on soil properties, soil functioning, and native plant diversity. Soil in mixed plots (those populated with all three invaders) contained higher levels of nitrifying bacteria, organic matter, nitrogen, and carbon as well as lower carbon to nitrogen ratio as compared to single species invaded plots and control plots. Furthermore, native plant diversity decreased with invasive plants co-occurrence. Differences in soil conditions and lower native plant diversity revealed the interactive potential of multiple invasive species in depleting biodiversity and eroding soil functionality, ultimately affecting ecological and biogeochemical processes both below and above ground. Our results highlight the need to prevent the impact of multispecies invasion, suggesting that riparian ecosystems affected by co-occurring invaders should be prioritized for invasion monitoring and ecological restoration.

## Introduction

One of the problems of globalization is biological invasion (Reaser et al., 2007). Trade-mediated dispersal of organisms beyond their natural range leads to the introduction and spread of invasive species that harm native biodiversity and impair different functions of socio-biological systems (Sala et al., 2000; Walsh et al., 2016; Qu et al., 2021). One definite consequence of increasing rates and volumes of such biotic exchange is the co-occurrence of multiple invasive species across different habitats (Kuebbing et al., 2013). Yet, research has mainly focused on the effects of single, individual species (Hulme et al., 2013; Kuebbing et al., 2013; Stricker et al., 2015; D’Antonio et al., 2017; Tekiela and Barney, 2017), although multispecies invasion is potentially more detrimental to ecosystems compared to single species invasion (Simberloff and Von Holle, 1999; Inderjit et al., 2005; Pisula and Meiners, 2010). Such a knowledge gap impairs our ability to understand the mechanisms underlying the potentially amplifying effects of multiple invasive species. Elucidating the impact of co-occurring invasive species has important ecological implications for management plans and conservation actions considering co-invaded ecosystems accretion.

Invasive plants modify soil conditions either directly by depositing leaf litter of different quality and quantity (Ehrenfeld, 2001), or indirectly by affecting microbial communities and their activity (Kourtev et al., 2003; Qu et al., 2021). When multiple invasive species co-occur within the same community, co-occurrence of invasive plants can be explained by their same or similar introduction pathway or by interspecific interactions (Simberloff and Von Holle, 1999; Richardson et al., 2000). Those interactions among invasive species often involve the above-mentioned modification of soil properties in such a way that it further increases the establishment, spread, and impact of other invasive species (Vitousek and Walker, 1989; Kuebbing and Nuñez, 2016). Looking at net effects, one invader can inhibit or facilitate another one (Flory and Bauer, 2014; Kuebbing and Nuñez, 2016; Zhang et al., 2020). Likewise, the overall impact of multiple invaders on native biodiversity and soil functioning may result from facilitative (positive) interactions, whereby multiple species increase the magnitude of their combined effects as compared to their individual effect, or from competitive (negative) interactions, in which case the combined effects are weaker than a single-species effect (Lortie et al., 2021). Interactions among multiple invaders can also be neutral, in which case their combined impact is negligible (Kuebbing, 2014). Generally, interactions among co-occurring invasive plants are more commonly negative or neutral, whereas positive interactions, although rare, are more common among woody plants and in communities with nitrogen fixing species (Kuebbing and Nuñez, 2015). Therefore, the co-occurrence of multiple invasive species makes restoration more complicated and has subsequent cascading effects on ecosystem functioning (Qu et al., 2021). Although there is ample evidence that single invasive species negatively affect soil processes and native plant communities, the linkages among multiple woody invaders, soil functionality and native plants remain overlooked. This is particularly the case for riparian ecosystems, which represent crucial habitats at the interface of terrestrial and aquatic environments.

The present study fills this gap in invasion biology by addressing the impact of three co-occurring invasive woody species on soil properties and native plant communities in the riparian zone. Being particularly prone to plant invasion (Pyšek and Prach, 1993), riparian habitats represent good model systems for studying ecological effects of multiple invasions (Planty-Tabacchi et al., 1996; Ehrenfeld and Stander, 2010). We answered the following research questions: (1) Does invasion impact increase with the co-occurrence of three invasive plants? (2) What are the differences in soil properties and native plant communities among non, single, and three-species invaded communities? (3) What are the direct and indirect relationships among invasive plants co-occurrence, native plant diversity and soil properties? We hypothesize that the impact of invasive plant species on soil properties and native plant communities increases with the number of invasive plant species.

## Materials and Methods

### Study Site

**FIGURE 1:**
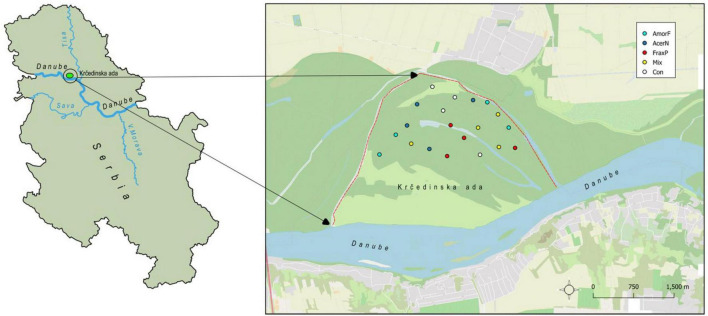
Location of the investigated riparian ecosystem and the distribution of plots across invasive species treatments (Con, control; AcerN, *A. negundo*; AmorF, *A. fruticosa*; FraxP, *F. pennsylvanica*; Mix, three-species mixture).

Our study was conducted at a riparian ecosystem located at *Krèedinska ada*, which is one of the largest river islands of the Danube River basin in Serbia (Figure 1). It is in the northern part of Serbia, Vojvodina Province, and is part of a larger floodplain complex and a Special Nature Reserve Koviljsko-Petrovaradinski Rit. The island has a history of invasion by the boxelder (*Acer negundo* L.), the green ash (*Fraxinus pennsylvanica* Marshall) and false indigo-bush (*Amorpha fruticosa* L.), which co-invade riparian areas of Eastern Europe and present major environmental management challenges. Their individual impact on ecosystems is rarely reported in the literature, while their combined impact is largely absent from available records. Their presence and co-occurrence in the surrounding floodplain area was first recorded by Parabućski (1972), implying a long history of species co-occurrence. According to Parabućski (1972), *F. pennsylvanica* and *A. negundo* were planted in the area at the same time, while *A. fruticosa* was probably introduced *via* the Danube River. Slavnić (1952) mentions *A. fruticosa* as a common species in riparian forests of willow stands in Vojvodina (ass. *Salicetum albae pannonicum*), which is the most common forest type in this floodplain complex.

The island was surveyed during May and June of 2014 by selecting four plots with *A. negundo* only (AcerN), four plots with *A. fruticosa* only (AmorF), four plots with *F. pennsylvanica* only (FraxP), four plots with all three invasive plants together (Mix), and four plots without invasive plants (Con). In *Mix* plots, the abundance of invaders was even (Supplementary Table 1), excluding the possibility of more abundant species having an advantage compared to less abundant ones. In total, 20 plots of 10 × 10 m were randomly placed within the same riparian ecosystem at a minimum distance of 300 m. All vascular plant species occurring in each plot were identified at the species level. The cover of herbaceous plants and invasive plant species (with a diameter at breast height of ≥5 cm) in each plot was visually estimated using the Braun Blanquet five-degree scale of abundance and dominance (Braun-Blanquet, 1964; Supplementary Table 1). All plots were at the same elevation and had similar soil texture (loam) and land use history (Figure 1). This way, all plots pose the same abiotic conditions, further minimizing the possibility that vegetation differed prior to species invasion. Species Accumulation Curve (Supplementary Figure 1) demonstrates that the sampling effort of 20 plots was exhaustive and robust enough to ensure a representative set of plant communities in the examined invaded ecosystem. The derivative of the fitted Species Accumulation Curve at the 20th plot was equal to 0.552, indicating that additional sampling would provide new relevant information on species richness, but rather that with 20 plots we captured the actual species pool.

### Soil Analyses

Soil was sampled with a 2.5 cm core diameter soil probe at 0-30 cm depth, three times per each plot. Samples were finally pooled per each plot. Each sample was air-dried and sieved to the <2 mm particle size, in accordance with ISO 11464 (2006). We analyzed the following soil biogeochemical properties: soil acidity (pH), calcium carbonate (CaCO_3_), organic matter (SOM), plant available phosphorus AL-P_2_O_5_ (AP), plant available potassium AL-K_2_O (AK), total nitrogen (N), carbon (C), carbon to nitrogen ratio (C:N), total sulfur (S), aluminum (Al), calcium (Ca), iron (Fe), potassium (K), magnesium (Mg), nitrifying bacteria (NB), and denitrifying bacteria (DB). For analyzing the nitrifying and DB, soil samples were collected aseptically from the top 30 cm layer using a hand shovel, taken to the laboratory on the same day and stored at 4°C. Prior to the analysis, soil samples were passed through a 2 mm sieve.

Given that all samples were of the same soil type (Supplementary Table 2), i.e., gleyic fluvisol (IUSS Working Group WRB, 2014), and had uniform characteristics, to validate soil texture gradient, particle size fractions were identified in 10 samples (Supplementary Table 2). Particle size distribution was determined in the <2 mm fraction using the pipette method (Van Reeuwijk, 2002), revealing presence of the following size fractions: (<2 μm), silt (2–20 μm), fine sand (20–200 μm), and coarse sand (200–2000 μm).

Soil pH was determined in water suspension using a glass electrode in accordance with the ISO 10390 (2005) methods. Calcium carbonate (CaCO_3_) content was determined in accordance with the ISO 10693 (1995) method for soil quality. SOM was measured by the Tjurin method, while the total nitrogen and carbon content was determined *via* elementary analysis (CHNSO VarioEL III) in accordance with the AOAC (2000) Official Method 972.43:2006. Readily available phosphorus P (AL) and readily available potassium K (AL) in soil were determined by ammonium lactate extraction (Egner et al., 1960). Detection of available P was performed spectrophotometrically at λ = 830 nm in a UV/VIS spectrophotometer using the phosphomolybdate-blue-method (Murphy and Riley, 1962), whereas available K was determined by ammonium lactate extraction (Egner et al., 1960) using flame photometer. The total content of micro and macro elements (Mg, Fe, S, Al, and Ca) in the soil samples was analyzed after digesting the soil in concentrated HNO_3_ and H_2_O_2_ (5 HNO_3_: 1 H_2_O_2_, and 1: 12 solid: solution ratio) by stepwise heating up to 180°C using a Milestone Vario EL III for 55 min. Elemental concentrations were determined by ICP-OES (Vista Pro-Axial, Varian) in accordance with the US EPA 200.7:2001 method. Quality control was periodically carried out with the IRMM BCR reference materials CRM-141R and CRM-142R. The recoveries were within 10% of the certified values.

The number of NB was determined in a liquid medium by inoculating suspensions of soil dilutions in test tubes with a medium of the following chemical composition: NaNO_2_ 10 g; K_2_HPO_4_ 0.5 g; NaCl 0.3 g; MgSO_4_ 0.5 g; MnSO_4_; Fe_2_(SO_4_)_3_; and distilled water 1000 ml. Samples were incubated for 4 days at 28°C, after which a few drops of reactive containing diphenylamine, distilled water and concentrated H_2_SO_4_ were added. The positive tubes had blue coloration. To determine the number of DB soil dilution suspensions were spread directly onto nutrient agar. Gil’tai medium was used to cultivate DB. After 48 h incubation at 28°C after which the reagents were poured over the medium and nitrate-reducing colonies were identified by red color. The reading was converted into 1 g of soil dry weight, i.e., the number of bacteria per 1 g of soil dry weight.

### Data Analysis

Data analysis was conducted in R 3.6.3 (R Core Team, 2020).

To answer the first question, we tested the differences in soil properties and plant communities among treatments (i.e., no invasive species or control, single invasive species, and three invasive species or mixture). We measured plant community structure as alpha diversity (i.e., species richness as well as Shannon index) following Oksanen et al. (2019). Then, we used generalized linear models (*glm* function in *base* package) with Normal distribution for soil parameters and Poisson distribution for plant species richness. This resulted in seventeen distinct univariate models. In each model, soil properties and plant diversity (richness) were the dependent, response variable and invasive species treatments were the independent, predictor variable (categorical, ordered factor, with control as the reference level). After evaluating model robustness (random residual distribution and q–q plots) and model fit, treatment significance was assessed in terms of parameter estimates (95% CI, *confint* function in *base* R package) as well as explained variance by means of ANOVA (Supplementary Table 3) (*Anova* function in *car* package; Fox and Weisberg, 2019). Invasive species treatment was also considered as a linear, numeric predictor. Results remained qualitatively the same (Supplementary Table 4), indicating the robustness of our findings.

To answer the second question, we tested the effects of invasive species treatments on (1) soil parameters, and (2) native plant diversity. We performed sparse partial least squares discriminant analysis (*splsda* function in *mixOmics* package; Rohart et al., 2017), which classifies plots depending on whole soil conditions and selects relevant variables of putative influence. This analysis was performed on the subset of soil variables that were of significant influence in the previous univariate analysis (i.e., SOM, N, C, C:N, NB). Then, to determine the significance of treatments, we extracted the loadings of the plots along the first two axes and adopted a linear model with loadings as the dependent variable and treatments as predictor (Legendre and Legendre, 2012). The significance of predictors was tested both in terms of model fit and 95% CI parameter estimates as well as in terms of explained variance using ANOVA (Fox and Weisberg, 2019). To visualize such multidimensional (i.e., multivariate) dataset, results were presented as a biplot.

Next, to answer the third question, we assessed the distribution of plant species depending on both soil conditions and invasive treatments by means of the canonical correspondence analysis (*cca* function in vegan; ter Braak, 1986; Legendre and Legendre, 2012; Oksanen et al., 2019). Here, the plant community matrix (i.e., plots in rows and plant species in columns with cover data as entries) was the response matrix while treatments and relevant soil properties (i.e., SOM, C:N, and NB) were fitted as constraining variables. Overall model significance was tested using ANOVA-like permutation test (*anova* function in *vegan*, Oksanen et al., 2019) and relationships between plant species and soil conditions as well as between plant species and invasive treatments were evaluated by looking at cca model loadings (*summary* function in *vegan*; Oksanen et al., 2019).

Finally, we assessed direct and indirect relations among native plants, soil properties and invasive species *via* structural equation modeling (SEM; Rosseel, 2012). We used the following SEM syntax: (i) regressions: (a) native plant species diversity (Shannon index) as a function of soil conditions and invasive species co-occurrence (i.e., the number of invasive plant species), and (b) soil conditions as a function of invasive species co-occurrence; (ii) latent variables: soil conditions (latent variable) as composed by the C:N ratio, SOM, N, C, and NB; and (iii) correlations: C:N ratio covarying with both N and C. This resulted in five regression parameters to be estimated. We used maximum likelihood estimation with robust bootstrapped SE and bootstrapped Chi-squared test statistics (i.e., Satorra–Bentler correction) for parameter estimation and model fitting evaluation (*sem* function in *lavaan*; Rosseel, 2012).

Although the sample size was *n* = 20, the structural equation model provided reliable results given that: (1) it ended normally and correctly converged after 38 iterations; (2) we did not overfit the number of model parameters as we remained with four degrees of freedom; (3) the model shows an acceptable performance since the *P*-value of the overall fit robust Chi-squared was equal to 0.149, indicating negligible discrepancy between the sample and fitted covariance matrices (i.e., the hypothesis of a perfect fit cannot be rejected); (4) likewise, the comparative fit index, which compares the fit of a target model to the fit of a null model and should be higher than 0.90, was in our case equal to 0.936; (5) the CI of root mean square error of approximation was 0.03–0.34, with *P*-value = 0.059; and (6) scaling factor for Satorra–Bentler correction was 1.269. Taken together, this output indicates that the results of the SEM are reliable.

## Results

### The Impact of Invasive Plants Increases With Their Co-occurrence

**FIGURE 2:**
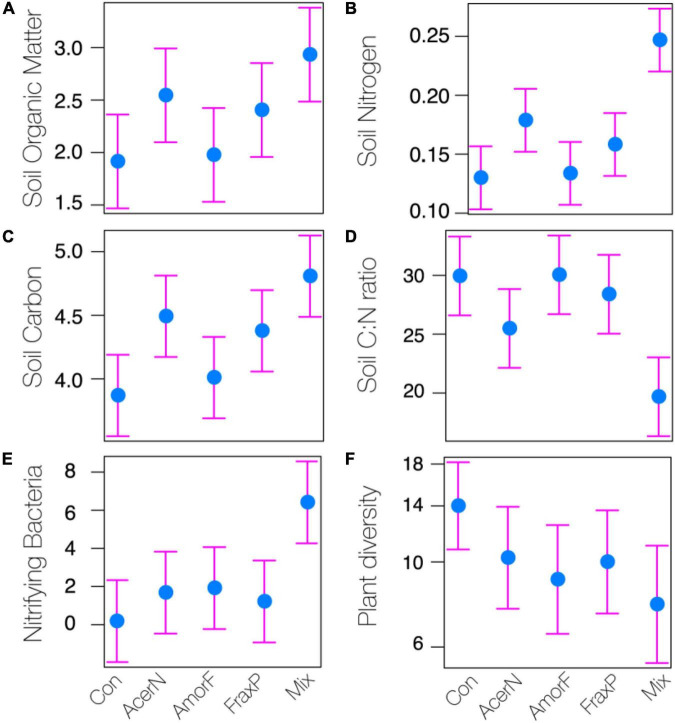
Effects of invasive species treatments (Con, control; AcerN, *A. negundo*; AmorF, *A. fruticosa*; FraxP, *F. pennsylvanica*; Mix, three-species mixture) on different soil parameters (**A:** organic matter; **B:** nitrogen; **C:** carbon; **D:** carbon to nitrogen ratio; **E:** nitrifying bacteria) and plant community (**F:** plant diversity). Estimated means and 95% CI are shown.

Our analysis revealed overall significant differences in SOM, N, C, C:N ratio, and NB among invasive species treatments (Figure 2), whereas pH, CaCO_3_, available P_2_O_5_, available K_2_O, total S, Al, Fe, Ca, K, and Mg, and DB showed less variation across treatments (Table 1).

**TABLE 1:** Summary of the ANOVA table for the regression model with invasive-species-treatment factor as predictor.

Response	Chi-square	*P*-value	*R* ^2^
pH	1.05	0.412	0.22
CaCO_3_	0.52	0.719	0.12
Humus	4.02	0.021	0.52
ALP_2_O_5_	1.22	0.343	0.25
ALK_2_O	1.51	0.25	0.29
N	14.42	0	0.79
C	6.32	0.003	0.63
CN	7.63	0.001	0.67
S	1.76	0.189	0.32
Al	0.21	0.932	0.05
Ca	0.21	0.929	0.05
Fe	0.57	0.69	0.13
K	0.2	0.937	0.05
Mg	0.93	0.471	0.2
NB	5.68	0.005	0.6
DB	1.26	0.33	0.25
Plant diversity	8.24	0.083	0.34

*Degrees of freedom for treatment and residuals were 4 and 19, respectively. For regression model estimates and 95% CI, see Supplementary Table 3.*

We report model parameter estimates with 95% CI and treatment significance in Supplementary Table 3. In particular, SOM significantly increased in *A. negundo* and *Mix* treatments as compared to control by 33 and 53%, respectively (Figure 2A), whereas 3 and 25% unsignificant differences were observed between *A. fruticosa* and *F. pennsylvanica* relative to control. N content significantly increased in *A. negundo* and *Mix* treatments as compared to control by 38 and 90%, respectively (Figure 2B), whereas 3 and 22% insignificant differences were observed between *A. fruticosa* and *F. pennsylvanica* relative to control. C content significantly increased in *A. negundo*, *F. pennsylvanica*, and *Mix* treatments as compared to control by 16, 13, and 24%, respectively, while 4% unsignificant differences were observed between *A. fruticosa* and control (Figure 2C). The C:N ratio marginally decreased by 15% and significantly decreased by 34% in *A. negundo* and *Mix* treatments as compared to control, respectively (Figure 2D). NB showed a significant sixfold increase in *Mix* treatments compared to control (Figure 2E). AlP_2_O_5_ significantly decreased by 68% in *A. negundo* as compared to control, while AlK_2_O marginally increased by 52% in *Mix* compared to control. Marginally significant differences were observed between *F. pennsylvanica* and control in S concentration (40% reduction). Finally, DB significantly decreased (by 81%) in *A. fruticosa* relative to control. All soil samples were found to be highly calcareous (above 20%) and slightly alkaline to alkaline, as their pH ranged from 8.02 to 8.28 (mean 8.17 ± 0.02 SE).

Finally, looking at plant diversity (i.e., the richness of native plant species), we found that invasive species treatments marginally affected the overall plant species diversity (*P* = 0.083; Table 1). Yet significant species-specific invasive effects were detected as plant diversity significantly declined in *A. fruticosa* and *Mix* treatments as compared to control by 17 and 22%, respectively (Figure 2F).

### Differences in Soil Properties and Native Plant Communities

**FIGURE 3:**
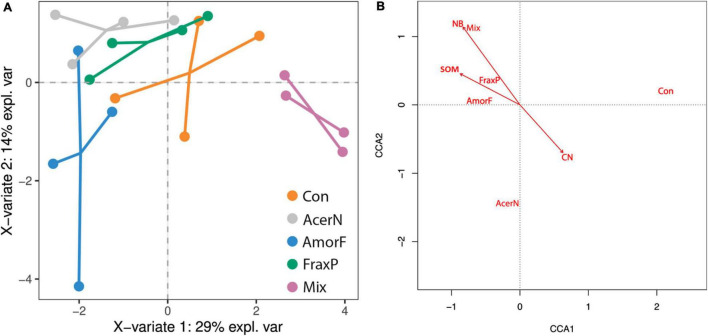
Multivariate relationships among invasive species co-occurrence, soil conditions, and plant communities. **(A)** Biplot with two main axes of variation (43% explained variance) in whole soil conditions (dots linking communities belong to the same treatment) in response to invasive treatments (orange: control; pink: *A. negundo*; blue: *A. fruticosa*; green: *F. pennsylvanica*; gray: Mix). **(B)** Biplot showing the effects of soil conditions (arrows) on plant species distribution (omitted for clarity) across invasive treatments (black text). The first two axes explain 30% of variance.

Multivariate relationships among invasive species co-occurrence, soil conditions, and plant communities are shown in Figure 3. The sPLS–DA results indicate that the first and second components explained 29 and 14% of the variance among variables and 25.0 and 24.9% of variance among plots (Figure 3A). In particular, N, C, NB, and SOM were positively correlated (with 0.47, 0.40, 0.38, and 0.37 correlation values, respectively), while C:N ratio was negatively correlated (a correlation of −0.43) with the first component. Moreover, Mg and CaCO_3_ were positively correlated (with 0.38 and 0.24 correlation values, respectively), while S, DB and AL-P_2_O_5_ were negatively correlated (−0.46, −0.46, and −0.34) with the second component.

The distribution of plots along the first component reflected the actual treatments (*R*^2^ = 0.80, *F*_4,15_ = 15.00, *P* < 0.001). The results yielded by the regression analysis with soil condition loadings and invasive treatments indicates that control was most negatively correlated with the first axis (β = −1.97 ± 0.54, *P* = 0.002; −3.11 to −0.83 95% CI), and this was the only statistically significant correlation, whereas *Mix* treatment emerged as the most differential and critical one (β = 5.28 ± 0.76, *P* < 0.001; 3.66–6.89 95% CI). The *F. pennsylvanica* (β = 1.52 ± 0.76, *P* = 0.063; −0.09 to 3.13 95% CI) and *A. negundo* (β = 2.46 ± 0.76, *P* = 0.005; 0.85–4.01 95% CI) single-species treatments were also significantly and marginally associated with the first axis, respectively, while *A. fruticosa* sites exhibited an inconsistent trend (β = 0.58 ± 0.76, *P* = 0.455; −1.03 to 2.19 95% CI).

### Direct and Indirect Relations Among Native Plants, Soil Properties, and Invasive Species

When looking at the multivariate response of plant communities to invasive species treatment accounting for differences in soil conditions, we found that soil conditions explained 57% of variance in plant species distribution across invasive treatments (*F*_7,12_ = 1.74, *P* < 0.001; Figure 3B). Sites were distributed following invasive treatments along the first axis (*P* < 0.001), with the control on one side (*x*_*control*_ = 1.89) and invasive species on the other (*x*_*acer*_ = −0.46, *x*_*amorpha*_ = −0.53, *x*_*fraxinus*_ = −0.50, *x*_*mix*_ = −0.60). Two constraining variables of SOM (*s* = −0.49) and NB (*s* = −0.47) were negatively correlated with this first axis, while C:N ratio (*s* = 0.35) was positively correlated with it.

The plant species most strongly associated with control were *Agropyron repens* (L.) P. Beauv., *Agrostis stolonifera* L., *Cynodon dactylon* (L.) Pers., *Diplotaxis muralis* (L.) DC., *Mentha pulegium* L., *Plantago media* L., *Plantago major* L., *Polygonum persicaria* L., *Rumex crispus* L., *Taraxacum officinale* (L.) Weber ex F.H.Wigg., *Trifolium repens* L., and *Xanthium spinosum* L. Species most strongly associated with *A. negundo* treatment were *Arctium lappa* L., *Gratiola officinalis* L., *Myosotis scorpioides* L., *Rorippa sylvestris* (L.) Besser, *Stachys palustris* L., and *Solanum dulcamara* L. Plant species most strongly associated with *Mix* treatment were *Polygonum hydropiper* L., *Rubus caesius* L., *Crataegus nigra* Waldst. & Kit., *Vitis riparia subsp. longii* W.R. Prince & Prince, and *Ulmus minor* Mill. (saplings).

**FIGURE 4:**
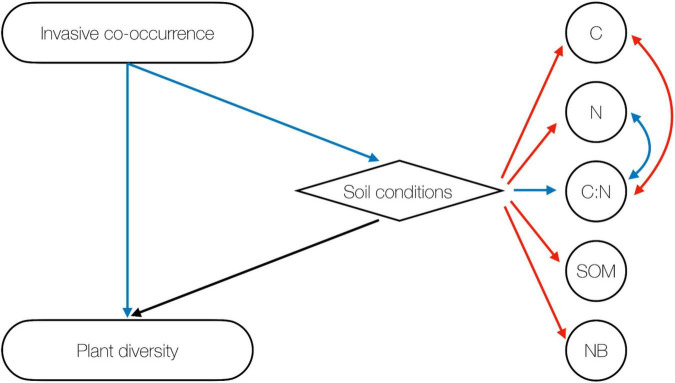
Structural equation modeling addressing the direct and indirect effects of invasive species co-occurrence on plant diversity. Blue arrows indicate negative, red arrows positive, and black arrows neutral effects.

Finally, we report the direct and indirect relations among invasive species co-occurrence, native plant diversity and soil conditions obtained by means of SEM (Figure 4). Results indicate that the co-occurrence of three invasive plants reduces native plant diversity (β = −0.96 ± 0.18, *P* < 0.001) and negatively impacts soil conditions (β = −1.01 ± 0.44, *P* = 0.022). Nevertheless, overall soil conditions had a neutral effect on plant diversity (β = 0.11 ± 0.43, *P* = 0.798). In other words, the invasion impact on native plant diversity and soil conditions is mainly direct and increases with increasing invasive species co-occurrence.

## Discussion

Multispecies invasion is an increasingly common phenomenon in ecosystems worldwide, but its impact has not been comprehensively explored yet. Furthermore, most of the research on biological invasions tended to focus on either above or below ground effects. Consequently, we still know little about how multiple invaders affect both below and above ground systems. Our paper bridges these knowledge gaps by addressing the ecological impact caused by multispecies invasion on plant and soil communities. We focused on three common co-invaders in Eastern Europe riparian sites and compared soil characteristics and native plant diversity among invaded communities. Our findings reveal that the influence of invasive plants is magnified by their co-occurrence. Furthermore, the direct and indirect relations among invasive species, plant, and soil communities suggest a mechanism by which facilitation among invasive plant species further amplifies the impact of biological invasions *via* plant–soil feedbacks.

### The Impact of Invasive Plants on Soil Properties

We report significant differences in soil properties and native plant diversity among invasive species treatments. Significant alterations are detected in the presence of single invasive species but were more pronounced with multiple species invasion. Simultaneous invasion and long-term co-occurrence of *A. negundo*, *F. pennsylvanica*, and *A. fruticosa* has been documented at the study site (Parabućski, 1972). Our results indicate their joint, enhanced impact on both above and below ground communities. Soils invaded by multiple plant species had significantly higher concentration of carbon, nitrogen, organic matter and NB, and significantly lower carbon to nitrogen ratio, compared to non-invaded soils and those invaded by single species.

Nitrogen content substantially increased in three species invaded communities as compared to single species invaded communities, with an increasing magnitude from *A. negundo* and *F. pennsylvanica* to *A. fruticosa* and control. Our results of similar total N content between *A. fruticosa* communities and control are in accordance with previous findings reported by Boscutti et al. (2020). These authors have also found increased nitrification in soils under *A. fruticosa*, which aligns with our findings of higher NB abundance in *A. fruticosa* soil communities. Looking at three invasive species co-occurrences, the significant nitrogen increase may be attributed to a higher leaf litter volume generated by *A. negundo*, *A. fruticosa*, and *F. pennsylvanica*, which decomposes at a higher rate compared to litter produced by native plants (Janušauskaitė and Straigyte, 2011; Krevš et al., 2013; Incerti et al., 2018; Reed, 2020). This is not a trait common to every plant species, but it is rather a characteristic of high-impact invaders, i.e., the strongest ecosystem modifiers (Jo et al., 2016).

A significant increase in NB in invaded communities was also noted by other authors in annual grasses soils (Hawkes et al., 2005; McLeod et al., 2016) as well as in shrubland soils (Coats, 2013). Although we did not measure ammonium and nitrate soil concentrations but solely total nitrogen, increase in the number of NB in multispecies invaded soils indicates greater concentrations and availability of these two nitrogen forms. This not only changes the soil microbial community structure and the belowground biota, but can also impact above ground communities. Indeed, higher nitrogen and NB make more competitive environments for native plants (Laungani and Knops, 2009; Heberling and Fridley, 2013) but facilitates the growth and spread of invasive plants (Jo et al., 2017), which can ultimately decrease plant diversity. Feedback relations between plant invaders and soil microbial organisms resulting in plants securing their N uptake, and ultimately fitness, is a negative tendency experimentally demonstrated for the nitrification process (Wolfe and Klironomos, 2005; Lee et al., 2012) that supports biological invasion (Jo et al., 2016). Such a trend can increase with current global changes associated with nitrogen deposition. Although NB abundance is a result of either plant–microbial interactions, or plant-mediated changes in soil properties, it remains to be established whether the increase of NB in the soil further drives species invasion and plant competitive exclusion.

The higher carbon content in three species invaded soils as compared to single species ones supports the general trend of invaders exerting direct influence on soil processes by affecting nutrient inputs through litter decomposition (Wardle et al., 2004; Liao et al., 2008; Lorenzo et al., 2010; Pyšek and Richardson, 2010; Vila et al., 2011; Si et al., 2013; Simberloff et al., 2013; Pereira and Ferreira, 2021). Those changes in soil carbon storage can be greatly enhanced by invader co-occurrence and could be due to a pronounced divergence in leaf and litter traits between native and invasive plants. Although we did not measure the physical and chemical leaf traits of examined species, which would also allow us to look at litter decomposition rates (Richardson et al., 2000), our results indicate that investigated invasive plants can exert a strong impact on ecosystems by modifying soil properties and nutrient cycling. Significant increase in carbon soil content at co-invaded communities may also lead to imbalance in natural soil carbon stock with long-term consequences for ecosystem processes. The markedly increased organic matter content in *Mix* plots could also be attributed to a greater production of plant biomass and the resulting higher decomposition rate. Furthermore, high carbon input generates more carbohydrates available to nitrogen fixing bacteria, which may result in increased nitrogen soil content (Knops et al., 2002).

As carbon and nitrogen are key macro elements, the remarkable influence of invasive plants on biogeochemical cycles affects overall ecosystem dynamics. Such impact is more pronounced under multiple, prolonged invasions that would render the site more prone to invaders (Ehrenfeld, 2001). A significantly higher increase in organic matter content in *Mix* communities, compared to single species invaded and control, confirms the magnified, synergistic impact of these invasive species on soil properties. Higher carbon, organic matter, and nitrogen content in *Mix* soils as compared to single invasive species communities may be ascribed to the facilitative effects among soil microbial communities, *A. negundo* and *F. pennsylvanica* (Jo et al., 2017). As plant height positively correlates with the aboveground biomass and leaf mass, we may deduce that *A. fruticosa*, being a shrub, contributes less than its tree neighbors (*A. negundo* and *F. pennsylvanica*) to soil nutrient inputs through the leaf litter decomposition pathway.

### The Impact of Invasive Plants on Native Plant Diversity

Although native plant diversity decreased in single species-invaded plots relative to controls, the reduction was much more pronounced in *Mix* plots, demonstrating greater impact of multiple invaders. The impact an invasive plant species exerts on native plant communities is closely related to its colonization rate and dominance in the invaded community (Richardson et al., 1989; Hejda and Pyšek, 2006; Chmura et al., 2015; Czarniecka-Wiera et al., 2019). In our study, both single and multispecies invaded plots contained similar invasive species cover, which is why the more pronounced native plant diversity reduction in *Mix* plant communities is not exclusively the result of invaders occupying more space, but rather suggests synergistic impacts of their co-occurrence. All three investigated invaders have strong allelopathic effects (Csizar, 2009), indicating the possibility that cumulative or non-additive allelopathic effects among *A. negundo*, *F. pennsylvanica*, and *A. fruticosa* may be partially responsible for native plant diversity reduction. Other known competitively superior traits of investigated invaders such as rapid growth, rapid vegetative regrowth, formation of dense clones and accumulation of thick litter layer, may have contributed to exclusion of native species such as *Salix alba*, and to significant reduction in herbaceous ground cover.

Unlike single invaded plots, where native diversity reduction is recorded only for herbaceous plant species, complete absence of *S. alba* from *Mix* plots suggests that combined effect of investigated invasive plants is more powerful in suppressing native woody plants. Native herbaceous plants that were locally lost after single and multiple include *A. repens*, *A. stolonifera*, *C. dactylon*, *D. muralis*, *P. major*, *P. media*, *P. persicaria*, *R. crispus*, *Solanum nigrum*, *T. officinale*, *T. repens*, *X. spinosum*. The loss of native plants as well as their presence in only one out of four communities among single species-invaded treatments suggests a prominent impact of single invasive species on native plant diversity. On the other hand, native woody shrubs *R. caesius* and *C. nigra*, which grow well in human-disturbed environments, had the highest cover in *Mix* plots. Likewise, presence of *U. minor* juveniles in *Mix* communities is in line with previous findings (Hejda, 2013) that juveniles of tree species tend to be more prevalent in the invaded vegetation and are less impacted by invasion than herbaceous plants.

Yet, our results do not align with previous findings of a much lower cumulative impact of multiple invaders on native plant diversity compared to single species invasion (Lenda et al., 2019). Such apparent inconsistency suggests that species-specific traits of invaders play a pivotal role in affecting native plant diversity. The impact of those traits also depends on native vegetation and ecosystem type. Indeed, Hulme and Bremner (2006) found that many native plants displaced by invaders in *Mix* plots are ruderal species. Although a negative relationship between native plant diversity and invasibility has been reported by other authors (Brown and Peet, 2003; Hulme and Bremner, 2006; Hejda et al., 2009), their investigations primarily focused on interactions between one invasive species and native vegetation. Thus, our results suggest that positive associations among fast-growing tree and shrub invasive species may increase the impact of invasion on native, ruderal plants.

### Direct and Indirect Relations Among Native Plants, Soil Properties, and Invasive Species

Correlative relationships among invasive plant co-occurrence, plant species diversity and soil, analyzed through SEM, are suggestive of causality among invasion and below–above ground communities. Results of this model indicate a negative direct, linear impact of invasive species co-occurrence on both native plant diversity and soil conditions. Even though we did not detect a direct link between native plant diversity and soil communities, differences in soil conditions explained 57% of the variance in plant species distribution. Notably, soil organic matter and NB were the most important predictors of vegetation patterns. This result reflects the strength of these two soil factors, modified by different invasive treatments, in affecting plant species distribution.

We conclude that the synergy among investigated invasive species may help secure their long-term persistence, which may accelerate soil modification and native plant species loss, while negatively affecting site restoration efforts. Indeed, our results may help in driving mitigation of multiple invasive species by directing future efforts on both above and below ground communities, besides directly treating invasive species. Further research with additional ecosystems and manipulation of species combinations and density, including different functional diversity levels of native species at various life-history stages, would help to determine impact thresholds and better understand mechanisms underlying invader interaction effects.

## Data Availability Statement

The original contributions presented in the study are included in the article/Supplementary Material, further inquiries can be directed to the corresponding authors.

## Author Contributions

DV: conception and design of the study, fieldwork, data collection, and writing. GL: data analysis, writing, and review. SM: soil analysis and interpretation. DM: supervision, literature search, and review. All authors revised the manuscript and gave final approval for publication.

## Conflict of Interest

The authors declare that the research was conducted in the absence of any commercial or financial relationships that could be construed as a potential conflict of interest.

## Publisher’s Note

All claims expressed in this article are solely those of the authors and do not necessarily represent those of their affiliated organizations, or those of the publisher, the editors and the reviewers. Any product that may be evaluated in this article, or claim that may be made by its manufacturer, is not guaranteed or endorsed by the publisher.
