# Supplementary material for: The Impact of Multiple Species Invasion on Soil and Plant Communities Increases With Invasive Species Co-occurrence
Source: Front Plant Sci. 2022 May 31;13:875824. doi: 10.3389/fpls.2022.875824 (PMC9194948; doi:10.3389/fpls.2022.875824)
Supplement: Supplementary file 1 [file Data_Sheet_1.docx]

**The impact of multiple species invasion on soil and plant communities increases with invasive species co-occurrence**

Vujanović Dušanka*, Losapio Gianalberto, Milić Stanko, Milić Dubravka

**BioSense Institute, University of Novi Sad, Dr Zorana Đinđića 1, Novi Sad 21000; Serbia; dusanka.vujanovic@biosense.rs*

**Supplementary Figure S1**


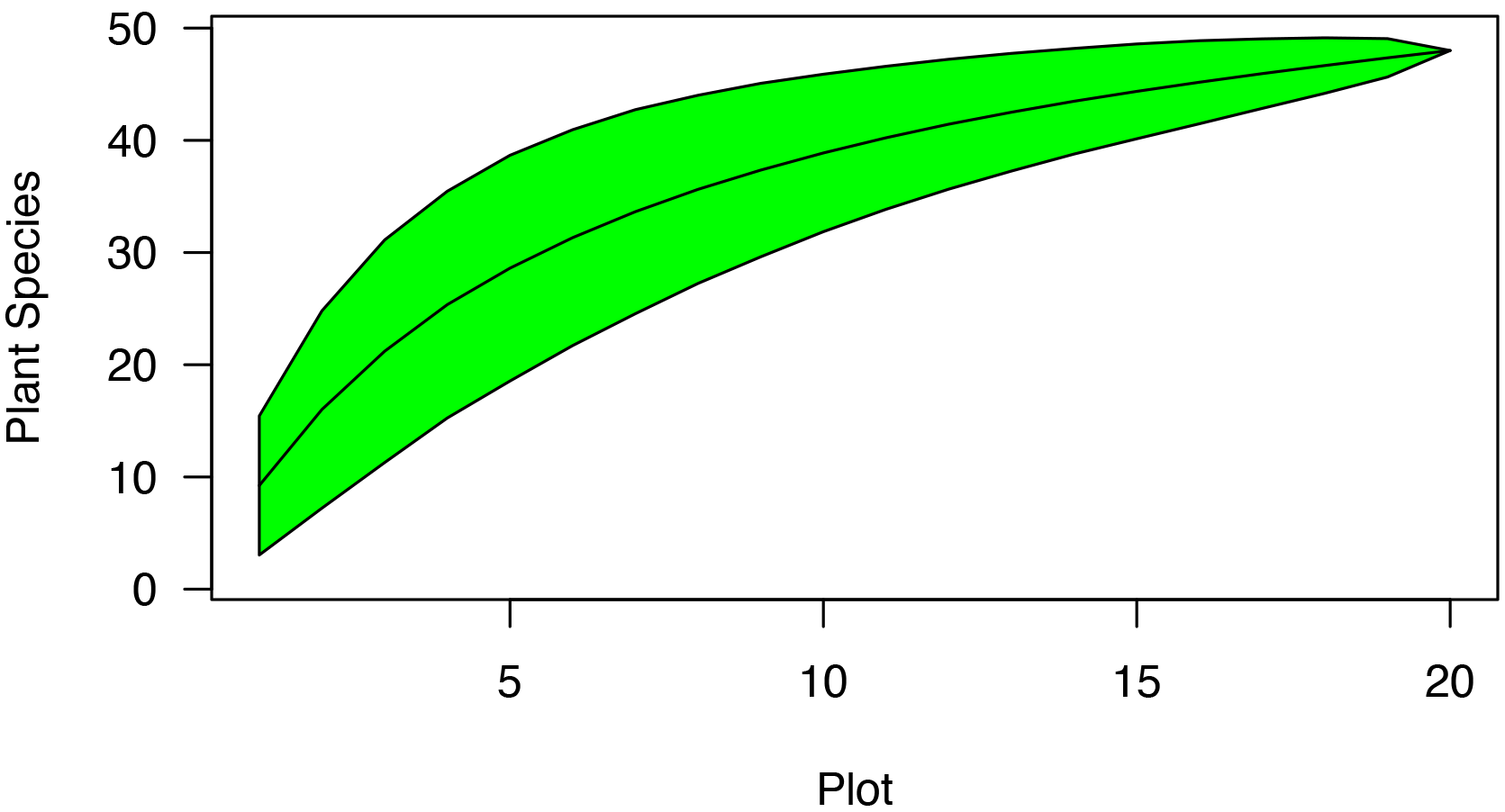


**Figure S1:** Species Accumulation Curve for the 20 samples plots (x-axis) demonstrates that we sampled a representative set of the species pool (y-axis) in the studied riparian ecosystem
