# Supplementary material for: The Impact of Multiple Species Invasion on Soil and Plant Communities Increases With Invasive Species Co-occurrence
Source: Front Plant Sci. 2022 May 31;13:875824. doi: 10.3389/fpls.2022.875824 (PMC9194948; doi:10.3389/fpls.2022.875824)
Supplement: Supplementary file 2 [file Table_1.docx]

**The impact of multiple species invasion on soil and plant communities increases with invasive species co-occurrence**

Vujanović Dušanka*, Losapio Gianalberto, Milić Stanko, Milić Dubravka

**BioSense Institute, University of Novi Sad, Dr Zorana Đinđića 1, Novi Sad 21000; Serbia; dusanka.vujanovic@biosense.rs*

**Supplementary Table S1**

| **Mean (± SE) value of percent cover of plant species for each group of treatments** | | | | | |
| --- | --- | --- | --- | --- | --- |
| Scientific name | Single species invaded plots *AcerN* | Single species invaded plots *AmorF* | Single species invaded plots  *FraxP* | Multispecies invaded plots Mix | Control  Con |
| ***Acer negundo****** | 37.5±7.2 |  |  | 37.5±7.2 |  |
| *Agropyron repens* |  |  |  |  | 18.75±6.2 |
| *Agrostis stolonifera* |  |  |  |  | 32.5±10.9 |
| *Amaranthus lividus* |  | 12.5±7.2 | 6.25±6.2 |  |  |
| ***Amorpha fruticosa****** |  | 62.5±7.2 |  | 43.75±11.9 |  |
| *Arctium lappa* | 7.5±5.9 |  |  |  |  |
| *Cardamine pratensis* |  |  | 6.25±6.2 |  |  |
| *Convolvulus arvensis* | 6.25±6.2 | 6.25±6.2 | 6.25±6.2 |  |  |
| *Crataegus nigra* | 6.25±6.2 | 12.5±7.2 | 26.25±9.2 | 37.5±7.2 |  |
| *Cynodon dactylon* |  |  |  |  | 62.5±7.2 |
| *Diplotaxis muralis* |  |  |  |  | 8.75±5.5 |
| *Euphorbia lucida* | 7.5±5.9 |  |  |  | 1.25±1.2 |
| *Festuca pratensis* |  | 12.5±12.5 | 7.5±5.9 |  |  |
| ***Fraxinus pennsylvanica****** |  |  | 37.5±12.5 | 31.25±6.2 |  |
| *Galium aparine* |  |  | 12.5±12.5 | 6.25±6.2 |  |
| *Galium palustre* | 7.5±5.9 | 12.5±12.5 |  |  |  |
| *Gratiola officinalis* | 18.75±18.7 |  | 12.5±12.5 |  |  |
| *Inula britannica* |  | 18.75±18.7 |  |  | 8.75±5.5 |
| *Lysimachia nummularia* | 12.5±12.5 | 25±10.2 | 18.75±11.9 | 12.5±12.5 |  |
| *Mentha aquatica* |  | 1.25±1.25 |  | 6.25±6.2 | 20±11.3 |
| *Mentha pulegium* |  |  |  |  | 7.5±5.9 |
| *Myosotis scorpioides* | 12.5±7.2 | 12.5±12.5 |  |  |  |
| *Plantago major* |  |  |  |  | 8.75±5.5 |
| *Plantago media* |  |  |  |  | 15±11.7 |
| *Polygonum hydropiper* | 6.25±6.2 |  | 6.25±6.2 | 18.75±11.9 | 25±14.4 |
| *Polygonum persicaria* |  |  |  |  | 8.75±5.5 |
| *Populus sp.* | 50±28.8 | 50±25 | 50±28.8 | 25±17.6 |  |
| *Potentilla anserina* | 12.5±12.5 |  | 12.5±12.5 |  | 5±0 |
| *Potentilla reptans* | 12.5±12.5 | 12.5±7.2 | 31.25±18.7 |  | 38.75±11.2 |
| *Rannunculus reptans* |  |  |  |  | 8.75±5.5 |
| *Rorippa sylvestris* | 18.75±11.9 |  |  |  |  |
| *Rubus caesius* | 68.75±15.7 | 43.75±25.7 | 75±10.2 | 87.5±7.2 |  |
| *Rumex acetosa* |  | 1.25±1.2 | 6.25±6.2 |  |  |
| *Rumex crispus* |  |  |  |  | 10±5 |
| *Salix alba* | 50±28.8 | 37.5±23.9 | 50±28.8 |  | 2.5±1.4 |
| *Sanguisorba officinalis* |  | 6.25±6.2 |  |  |  |
| *Solanum dulcamara* | 6.25±6.2 |  |  |  |  |
| *Solanum nigrum* |  |  |  |  | 2.5±1.4 |
| *Stachys palustris* | 12.5±12.5 |  |  |  |  |
| *Taraxacum officinale* |  |  |  |  | 8.75±5.5 |
| *Trifolium repens* |  |  |  |  | 25±10.2 |
| *Typha latifolia* | 6.25±6.2 | 6.25±6.2 | 7.5±5.9 | 6.25±6.2 |  |
| *Ulmus minor* |  |  |  | 13.75±6.5 |  |
| *Veronica catenata* |  |  | 6.25±6.2 |  |  |
| *Vitis riparia subsp. longii* | 6.25±12.5 | 18.75±11.9 | 18.75±11.9 | 37.5±16.1 |  |
| *Xanthium spinosum* |  |  |  |  | 3.75±1.2 |

**Table S1:** Mean (± SE) value of percent cover of plant species for each group of treatments (AcerN, AmorF, FraxP, Mix, Con). Means are based on four plots within each group. Invasive plants are accentuated with bold letters and asterisk.
