# Supplementary material for: The Impact of Multiple Species Invasion on Soil and Plant Communities Increases With Invasive Species Co-occurrence
Source: Front Plant Sci. 2022 May 31;13:875824. doi: 10.3389/fpls.2022.875824 (PMC9194948; doi:10.3389/fpls.2022.875824)
Supplement: Supplementary file 3 [file Table_2.docx]

**The impact of multiple species invasion on soil and plant communities increases with invasive species co-occurrence**

Vujanović Dušanka*, Losapio Gianalberto, Milić Stanko, Milić Dubravka

**BioSense Institute, University of Novi Sad, Dr Zorana Đinđića 1, Novi Sad 21000; Serbia; dusanka.vujanovic@biosense.rs*

**Supplementary Table S2**

| Sample | Coarse sand % | Fine sand % | Silt % | Clay % | Texture class |
| --- | --- | --- | --- | --- | --- |
| 1 | 1,94 | 44,10 | 39,72 | 14,24 | Loam |
| 2 | 2,89 | 44,27 | 38,84 | 14,00 | Loam |
| 3 | 1,07 | 68,53 | 23,20 | 7,20 | Sandy loam |
| 4 | 1,98 | 49,69 | 32,04 | 11,84 | loam |
| 5 | 2,35 | 43,77 | 40,48 | 13,40 | loam |
| 6 | 1,01 | 54,59 | 34,32 | 10,08 | loam |
| 7 | 1,48 | 58,16 | 30,28 | 10,08 | loam |
| 8 | 2,87 | 52,41 | 34,32 | 10,40 | loam |
| 9 | 2,16 | 58,24 | 30,72 | 12,88 | loam |
| 10 | 2,23 | 44,20 | 38,37 | 14,17 | loam |

**Table S2: Soil texture of analyzed soil samples**
