# Supplementary material for: The Impact of Multiple Species Invasion on Soil and Plant Communities Increases With Invasive Species Co-occurrence
Source: Front Plant Sci. 2022 May 31;13:875824. doi: 10.3389/fpls.2022.875824 (PMC9194948; doi:10.3389/fpls.2022.875824)
Supplement: Supplementary file 4 [file Table_3.docx]

**The impact of multiple species invasion on soil and plant communities increases with invasive species co-occurrence**

Vujanović Dušanka*, Losapio Gianalberto, Milić Stanko, Milić Dubravka

**BioSense Institute, University of Novi Sad, Dr Zorana Đinđića 1, Novi Sad 21000; Serbia; dusanka.vujanovic@biosense.rs*

**Supplementary Table S3**

|  | ***Control*** | | | ***AcerN*** | | | ***AmorF*** | | | ***FraxP*** | | | ***Mix*** | | |
| --- | --- | --- | --- | --- | --- | --- | --- | --- | --- | --- | --- | --- | --- | --- | --- |
|  | ***β*** | ***lower*** | ***upper*** | ***β*** | ***lower*** | ***upper*** | ***β*** | ***lower*** | ***upper*** | ***β*** | ***lower*** | ***upper*** | ***β*** | ***lower*** | ***upper*** |
| **pH** | 8.23 | 8.12 | 8.33 | -0.03 | -0.18 | 0.13 | -0.05 | -0.2 | 0.1 | -0.13 | -0.29 | 0.02 | -0.03 | -0.18 | 0.12 |
| **CaCO_3_** | 21.53 | 19.31 | 23.74 | 1.66 | -1.48 | 4.79 | 0.6 | -2.53 | 3.73 | 1.14 | -1.99 | 4.27 | -0.12 | -3.25 | 3.01 |
| **SOM** | 1.92 | 1.47 | 2.36 | 0.63 | 0 | 1.26 | 0.06 | -0.57 | 0.69 | 0.49 | -0.14 | 1.12 | 1.02 | 0.39 | 1.65 |
| **ALP_2_O_5_** | 15.57 | 8.22 | 22.93 | -10.57 | -20.98 | -0.17 | -6.85 | -17.26 | 3.56 | -5.5 | -15.91 | 4.91 | -6.62 | -17.03 | 3.78 |
| **ALK_2_O** | 11.25 | 6.32 | 16.18 | -0.23 | -7.2 | 6.75 | -0.32 | -7.3 | 6.65 | -1.02 | -8 | 5.95 | 5.9 | -1.07 | 12.87 |
| **N** | 0.13 | 0.1 | 0.16 | 0.05 | 0.01 | 0.09 | 0 | -0.03 | 0.04 | 0.03 | -0.01 | 0.07 | 0.12 | 0.08 | 0.15 |
| **C** | 3.87 | 3.55 | 4.19 | 0.62 | 0.17 | 1.07 | 0.14 | -0.31 | 0.59 | 0.51 | 0.06 | 0.96 | 0.94 | 0.49 | 1.39 |
| **C:N** | 29.94 | 26.59 | 33.29 | -4.45 | -9.19 | 0.28 | 0.1 | -4.63 | 4.83 | -1.55 | -6.28 | 3.18 | -10.26 | -14.99 | -5.53 |
| **S** | 0.06 | 0.04 | 0.08 | 0 | -0.03 | 0.02 | -0.02 | -0.05 | 0 | -0.01 | -0.04 | 0.01 | 0 | -0.02 | 0.03 |
| **Al** | 18955 | 14917.35 | 22992.65 | 702.5 | -5007.6 | 6412.6 | -860 | -6570.1 | 4850.1 | 20 | -5690.1 | 5730.1 | 1430 | -4280.1 | 7140.1 |
| **Ca** | 37685 | 35456.19 | 39913.81 | 800 | -2352.01 | 3952.01 | 232.5 | -2919.51 | 3384.51 | 117.5 | -3034.51 | 3269.51 | -535 | -3687.01 | 2617.01 |
| **Fe** | 20550 | 19106.26 | 21993.74 | 672.5 | -1369.25 | 2714.25 | 860 | -1181.75 | 2901.75 | 555 | -1486.75 | 2596.75 | 1410 | -631.75 | 3451.75 |
| **K** | 3722 | 2654.54 | 4789.46 | -118.5 | -1628.11 | 1391.11 | -514.5 | -2024.11 | 995.11 | -174.5 | -1684.11 | 1335.11 | 49.5 | -1460.11 | 1559.11 |
| **Mg** | 14880 | 14079.06 | 15680.94 | 837.5 | -295.2 | 1970.2 | 752.5 | -380.2 | 1885.2 | 802.5 | -330.2 | 1935.2 | 840 | -292.7 | 1972.7 |
| **NB** | 1844 | -19632.38 | 23320.38 | 14952.5 | -15419.69 | 45324.69 | 17342.25 | -13029.94 | 47714.44 | 10307.75 | -20064.44 | 40679.94 | 62272 | 31899.81 | 92644.19 |
| **DB** | 1627805 | 687618 | 2567992 | -572665 | -1902290.2 | 756960.2 | -1321387.5 | -2651012.7 | 8237.7 | -958002.5 | -2287627.7 | 371622.7 | -865872.25 | -2195497.45 | 463752.95 |
| **Plant diversity** | 2.64 | 2.37 | 2.89 | -0.31 | -0.72 | 0.09 | -0.44 | -0.87 | -0.03 | -0.34 | -0.75 | 0.07 | -0.59 | -1.04 | -0.16 |

**Table S3:** Summary of regression model of soil conditions (rows) in response to invasive species treatments (columns). Model parameters are indicated as *β* and 95% CI estimates as lower confidence level (*lci*) and upper confidence level (*uci*). Treatments are Control (*Con*), *A. negundo* (*AcerN*), *A. fruticosa* (*AmorF*), *F. pennsylvanica* (*FraxP*), and mix (*Mix*).
