# Supplementary material for: The Impact of Multiple Species Invasion on Soil and Plant Communities Increases With Invasive Species Co-occurrence
Source: Front Plant Sci. 2022 May 31;13:875824. doi: 10.3389/fpls.2022.875824 (PMC9194948; doi:10.3389/fpls.2022.875824)
Supplement: Supplementary file 5 [file Table_4.docx]

**The impact of multiple species invasion on soil and plant communities increases with invasive species co-occurrence**

Vujanović Dušanka*, Losapio Gianalberto, Milić Stanko, Milić Dubravka

**BioSense Institute, University of Novi Sad, Dr Zorana Đinđića 1, Novi Sad 21000; Serbia; dusanka.vujanovic@biosense.rs*

**Supplementary Table S4**

| **Response** | **p-value** | **estimate** | **Lower CI** | **Upper CI** |
| --- | --- | --- | --- | --- |
| pH | 0.917 | 0 | -0.05 | 0.05 |
| CaCO3 | 0.69 | -0.19 | -1.15 | 0.78 |
| Humus | 0.003 | 0.33 | 0.13 | 0.54 |
| ALP2O5 | 0.357 | -1.53 | -4.93 | 1.87 |
| ALK20. | 0.036 | 2.28 | 0.16 | 4.39 |
| N | 0 | 0.04 | 0.03 | 0.05 |
| C | 0.001 | 0.3 | 0.14 | 0.45 |
| CN | 0 | -3.6 | -5.2 | -2 |
| S | 0.533 | 0 | -0.01 | 0.01 |
| Al | 0.507 | 541.98 | -1139.75 | 2223.71 |
| Ca | 0.582 | -248.54 | -1181.11 | 684.02 |
| Fe | 0.138 | 441.77 | -155.66 | 1039.21 |
| K | 0.81 | 52.21 | -396.77 | 501.19 |
| Mg | 0.215 | 215.31 | -136.51 | 567.14 |
| Nitrifying bacteria | 0 | 21576.9 | 12544.95 | 30608.84 |
| Denitrifying bacteria | 0.333 | -205866.47 | -640163.78 | 228430.85 |
| Plant diversity | 0.011 | -0.19 | -0.35 | -0.04 |

**Table S4:** Summary of regression model with invasive species richness as a linear predictor
